# Alcohol and energy drinks: individual contribution of common ingredients on ethanol-induced behaviour

**DOI:** 10.3389/fnbeh.2023.1057262

**Published:** 2023-02-14

**Authors:** Ernesto Tarragon

**Affiliations:** Basic and Applied Psychobiology, Faculty of Psychology, Universidad Internacional de La Rioja, La Rioja, Spain

**Keywords:** ethanol, energy drinks (EDs), AMED, alcohol mixed with energy drinks, ethanol-induced behaviours

## Abstract

Since energy drinks (EDs) were sold to the general public as soft drinks and recreational beverages, mixing EDs with ethanol has grown in popularity, particularly among younger people. Given the research that links these drinks with higher risk behaviors and increased ethanol intake, ethanol combined with EDs (AmEDs) is a particularly worrying combination. EDs generally commonly include a variety of ingredients. Sugar, caffeine, taurine, and B-group vitamins are almost always present. Studies on the combined effect of ethanol and sugar and caffeine on ethanol-induced behaviors are extensive. Not so much in regards to taurine and vitamins. This review briefly summarises available information from research on the isolated compounds on EtOH-induced behaviors first, and secondly, the combination of AmEDs on EtOH effects. The conclusion is that additional research is needed to fully comprehend the characteristics and consequences of AmEDs on EtOH-induced behaviors.

## 1 Introduction

Mixing ethanol (EtOH) with energy drinks became popular several years ago (Weldy, 2010; Patrick et al., 2018). The goal of this combination is to maintain EtOH intake while sustaining high energy levels, thus limiting the narcotic effects of this alcohol. However, these drinks are usually packed with many ingredients, whose interaction, and their interaction with EtOH, has not been extensively tested.

The limited evidence exploring EtOH-energetic drinks seems to agree that this combination is a particularly risky one. Concretely, the consumption of these drinks is associated with increased risk behaviors and greater EtOH consumption, particularly among young individuals (O’Brien et al., 2008; Curran and Marczinski, 2017; Scalese et al., 2017; Vieno et al., 2018). Understanding the interaction between these beverages is key to advance in proposing preventive strategies towards the harmful effects of alcohol mixed with energy drinks (AmEDs). This review will resume relevant findings of studies exploring behavioral and cellular effects on the interaction between EtOH and common ingredients found in popular energy drinks: sucrose, caffeine, B-group vitamins, and taurine. Finally, it will review experimental work using energy drinks as a whole compound.

## 2 Behavioral and neurophysiological effects of EtOH intake

When chronically consumed, EtOH is a drug capable of producing many harmful effects both centrally and peripherally (Li et al., 2010). For instance, it is known that chronic ethanol metabolism in the liver causes fatty liver and general metabolic impairment and could result in chronic inhibition of AMPK activation to activate regulatory processes, such as gene expression, that take longer to initiate and reverse (Wilson and Matschinsky, 2020). In the central nervous system, EtOH alters the metabolism of neurons and astrocytes in the brain, which has an immediate impact on all aspects of neurotransmitter function release, receptor sensitivity, transport, and metabolism, as well as long-term changes in the cellular machinery (Wilson and Matschinsky, 2020). However, the chemical mechanism by which this alcohol exerts its effects on behaviour is only partially known.

There is robust evidence that the first metabolite of EtOH, acetaldehyde, mainly mediates the effects described above. This molecule plays a significant role in the behavioral effects of EtOH consumption, including hangover symptoms and liver damage (Wang et al., 2010) and the development of tolerance and dependence (Deng and Deitrich, 2008). Moreover, data shows that acetaldehyde enhances the rewarding effects of EtOH (Hipólito et al., 2007; Correa et al., 2012), leading to continued use and addiction. Additionally, acetaldehyde can contribute to the adverse effects of EtOH consumption, such as hangover symptoms and liver damage.

## 3 EtOH and sucrose

Alcoholic beverages are usually combined with soft drinks high in sugar (Samson et al., 1996). Early studies show that combining EtOH with sugar and other sweet molecules like fructose has a metabolic impact on EtOH’s pharmacokinetics, increasing EtOH blood clearance (Rawat, 1977). The predominant theory explaining the reason to mix sweet compounds with EtOH proposes that sugar and other sweeteners increase drinking by improving the taste of EtOH. Notably, Lemon et al. pointed to sucrose receptors as potential receptors for EtOH, given their findings on similar across-neurone response patterns to EtOH and sucrose (Lemon et al., 2004). This result suggests that both EtOH and sucrose are represented similarly by gustatory activity in the brain.

Consistent with this, it is known that several neural circuits responding to EtOH and sugar overlap (Barson and Leibowitz, 2016). Evidence shows a synergic effect of sucrose and EtOh on the activation of the nucleus accumbens shell, a region closely related to motivated behaviour (Czachowski and Samson, 2002; Stratford and Wirtshafter, 2011). Also, abundant preclinical work shows that, when binged on, sucrose can increase dopaminergic signalling to the nucleus accumbens in a similar fashion to what EtOH does (Rada et al., 2005; Rodríguez-Ortega et al., 2019).

## 4 EtOH and caffeine

The effects of caffeine on alcohol consumption have been extensively discussed (see López-Cruz et al., 2013 for a review). Some authors suggest that the enhanced arousal and subjective feeling of vigor brought on by caffeine may be the cause of the EDs’ facilitation of EtOH ingestion (Doggett et al., 2019; Oh et al., 2019). Caffeine and EtOH have complicated interactions in various domains. For instance, acute caffeine delivery appears to have the ability to enhance the stimulant effects of acute EtOH dosages at modest concentrations. However, a potentiation of the suppressive effects of both compounds is most noticeable at greater dosages of either caffeine or EtOH. On the other hand, it does not appear that continuous administration of either alters the acute levels at which locomotion can be induced.

Acute, low dosages of caffeine can counteract the effects of EtOH’s incoordination. However, high doses can intensify them. Interestingly, compared to A2A receptors, adenosine A1 receptors seem more crucial for these actions. Caffeine’s capacity to slow down the quick tolerance to EtOH’s effects on coordination has also been linked more to A1 than A2A receptors. However, there is a discrepancy in the literature. The specific effects of adenosine antagonism on EtOH self-administration may vary depending on conditions like dietary restriction, sexual activity, EtOH intake or reinforcement paradigms, or others. For instance, it has been proposed that the restricting effect of caffeine on EtOH intake may be due to high, intoxicating dosages of caffeine (Potthoff et al., 1983; Dietze and Kulkosky, 1991). However, the fact that prolonged caffeine prevented the impact of EtOH deprivation raises the possibility that caffeine may be effective in treating protective abstinence, but further research is needed to make this determination.

## 5 EtOH and B-vitamins

Vitamins are among the common ingredients added to energy drinks. Briefly, B2, B3, B5, B6, and B12 are the most frequent. In a recent study, Zielińska et al. (2022) analyzed the composition of various energy drinks. The authors report that vitamin B3 (niacinamide) content varied from 3.4 to 9.7 mg per 100 ml, with a higher amount usually found than that reported on the label. Another study analyzed 20 EDs and shots and concluded that vitamins B3, B6, and B12 were the most common; all of them well above the recommendations of daily value intake (Jagim et al., 2022).

Niacin has positive effects in restoring a healthy lipid profile and delaying the progression of atherosclerosis (Julius, 2015). It has been used for more than half a century in the treatment of lipid disorders, such as abnormally elevated concentrations of LDL, non-HDL cholesterol, triglycerides, and lipoproteins, and low concentrations of HDL (Julius, 2015; Garg et al., 2017).

EtOH consumption impairs vitamin function, especially B1, B2, B6, and B12 (Gibson et al., 2008; Miyazaki et al., 2012). However, some evidence suggests that a sufficient vitamin mixture in the diet could limit the negative effect of EtOH on B-vitamin metabolism (Miyazaki et al., 2012).

Unfortunately, the range variation of the different amounts of vitamins, together with the lack of behavioral studies on the combination of specific vitamins and EtOH, makes it extremely difficult to elucidate the individual or combined effect of these molecules on EtOH-induced behaviors.

## 6 EtOH and taurine

Taurine interacts mainly with glutamatergic and GABAergic receptors. According to *in vitro* research (Suárez and Solís, 2006), taurine activates presynaptic NMDA receptors at physiological levels while reducing glycine affinity for this receptor. One of the suggested explanations for the decrease in neuronal excitability brought on by taurine is this indirect suppression of NMDA signalling. Taurine’s interaction with the GABA system is one more way that it causes its inhibitory effects. It has been suggested that taurine functions as a positive allosteric modulator of the GABAA receptors’ alpha-2 subunit (Hansen et al., 2020). It is interesting to note that it has been proposed that EtOH acts on the same receptor.

Some studies suggest that injection of EtOH elevates taurine concentrations both centrally (brain) and peripherally (circulating blood). For instance, animal models show that taurine exerts protective effects by acting as an antioxidant, mainly by increasing glutathione and peroxide dismutase levels in the liver (Yildiz et al., 2019). Also, there is evidence that oral administration of taurine prevents the development of EtOH-induced hypertension in rats (Harada et al., 2000). Centrally, taurine is released in many alcoholism-related brain regions after both chronic and acute EtOH administration. Acute EtOH treatment in particular (0.5, 1, 2, and 3 g/kg, i.p.) enhances taurine release in the amygdala (Quertemont et al., 1998, 1999). On the other hand, voluntary ingestion of EtOH at 10% concentration stimulates taurine release in the nucleus accumbens (nAcc; Li et al., 2010). Contrary to this, other research demonstrates both prolonged and acute injection of EtOH lowers taurine levels in the brain (Iwata et al., 1980). Iwata et al. (1980) however, focused on the cerebellum, brainstem, and prefrontal cortex. This discrepancy should be taken into account when considering all the results.

Using the two-bottle choice paradigm, Pulcinelli et al. (2020) show that chronic taurine treatment (100 mg/kg, i.p.) increases rats’ voluntary alcohol intake and preference. Additionally, the rats subjected to chronic EtOH ingestion (20% w/v) showed anxiolytic effects. According to prior studies, the authors hypothesize that taurine’s anxiolytic effects may be caused by interactions with the GABA and DA systems. These results are relevant in view of the potential impact of taurine in AmEDs. In addition, taurine may augment the increased EtOH consumption linked to AmEDs in these beverages (Messiha, 1979). However, in more recent research on the interaction between taurine and EtOH, the co-treatment of taurine and EtOH did not have any discernible effects on ataxia, loss of the righting reflex, or locomotor activity. Only one strain of mice was used for the study (C57BL/6J mice), and taurine supplementation alone only marginally improved locomotor activity during the first five minutes following injection. This shows that genetics may affect how people react to taurine and may be a topic to investigate when thinking about AmEDs. Ulenius et al. recently confirmed these findings. The authors found no difference in the animals given with vehicle vs. taurine at 30, 60, 300, or 600 mg/kg (i.p.) in terms of their ability to move (Ulenius et al., 2019). For a review of the behavioral and cognitive effects of taurine on AmEDs mixed with EtOH, see Tarragon et al. (2021).

## 7 EtOH and energy drinks

Energy drinks provide immediate access to relatively high doses of caffeine and raise the possibility of clinically significant interactions between caffeine and EtOH when combined. There is a considerable amount of evidence connecting AmEDs to higher levels of alcohol consumption (Marczinski and Fillmore, 2014; Asorey et al., 2018; Doggett et al., 2019). Most of the research indicates that overall EtOH consumption at the end of a drinking session increases when caffeinated beverages add to the mix (Simon and Mosher, 2007; Verster et al., 2018). It has also been demonstrated that AmED, as opposed to EtOH alone, encourages a higher conditioned response to environmental signals linked with EtOH (Takahashi et al., 2015).

In a recent study, it was shown that rats are capable of self-administrating an energy drink called Red Bull^®^ that contains sucrose (11%), taurine (0.4%), and caffeine (0.32%). Additionally, it is shown that Red Bull^®^ (RB) + EtOH self-administration is linked to drinking alcohol in larger quantities, resulting in greater levels of EtOH in the blood (Roldán et al., 2018). This highlights the danger that AmED may pose in encouraging the development of addictive behaviors (May et al., 2015).

In another recent study, the likelihood of consuming AmEDs shortly nearly doubles if a person recently took non-alcoholic EDs (Doggett et al., 2019). Some of the chemicals found in EDs may improve EtOH’s reinforcing characteristics, lessen the aversive effects it causes, or both (Roldán et al., 2018). These data demonstrate the potential value of pharmacological substances other than caffeine in investigating AmEDs. Hence the importance of understanding the individual effects of these various chemicals.

## 8 Conclusion

Evidence shows that AmEDs are especially harmful during critical developmental periods, like adolescence and the prenatal phases (Brown et al., 2008; Squeglia et al., 2009; Al-Basher et al., 2018). Significantly, the consumption of AmEDs is increasing among pregnant women, who are mostly unaware of the potentially harmful effects on their unborn offspring, sometimes carrying over into puberty (Alamneh et al., 2020; Lees et al., 2020). According to numerous research, the primary motivations for combining EDs with EtOH, are to mask EtOH’s flavor, raise arousal levels, and quicken drunkenness (Peacock et al., 2015; Verster et al., 2018; Doggett et al., 2019). There is a lot of information on the interactions between EtOH and sucrose (Sharpe and Samson, 2003; Avena et al., 2004; Dorofeikova et al., 2017), as well as EtOH and caffeine (Kunin et al., 2000; Ferré and O’Brien, 2011; López-Cruz et al., 2013). However, studies addressing the potential impact of other common ingredients in these drinks are surprisingly rarer. This review hopes that it will be helpful not only to researchers looking for a place to start when developing studies focused on the voluntary use of EtOH and EDs but also to be of interest to the younger and pregnant population of potential risks these beverages imply.

## Author contributions

This manuscript was entirely written by the listed author.

## Conflict of Interest

The authors declare that the research was conducted in the absence of any commercial or financial relationships that could be construed as a potential conflict of interest.

## Publisher’s Note

All claims expressed in this article are solely those of the authors and do not necessarily represent those of their affiliated organizations, or those of the publisher, the editors and the reviewers. Any product that may be evaluated in this article, or claim that may be made by its manufacturer, is not guaranteed or endorsed by the publisher.

## References

[B1] Al-Basher G. I. Aljabal H. Almeer R. S. Allam A. A. Mahmoud A. M. (2018). Perinatal exposure to energy drink induces oxidative damage in the liver, kidney and brain and behavioral alterations in mice offspring. Biomed. Pharmacother. 102, 798–811. 10.1016/j.biopha.2018.03.139 29605768

[B2] Alamneh A. A. Endris B. S. Gebreyesus S. H. (2020). Caffeine, alcohol, khat and tobacco use during pregnancy in Butajira, South Central Ethiopia. PLoS One 15:e0232712. 10.1371/journal.pone.0232712 32384102 PMC7209255

[B3] Asorey L. G. Carbone S. Gonzalez B. J. Cutrera R. A. (2018). Behavioral effects of the combined use of alcohol and energy drinks on alcohol hangover in an experimental mice model. Neurosci. Lett. 670, 1–7. 10.1016/j.neulet.2018.01.030 29355695

[B4] Avena N. M. Carrillo C. A. Needham L. Leibowitz S. F. Hoebel B. G. (2004). Sugar-dependent rats show enhanced intake of unsweetened ethanol. Alcohol 34, 203–209. 10.1016/j.alcohol.2004.09.006 15902914

[B6] Barson J. R. Leibowitz S. F. (2016). Hypothalamic neuropeptide signaling in alcohol addiction. Prog. Neuropsychopharmacol. Biol. Psychiatry 65, 321–329. 10.1016/j.pnpbp.2015.02.006 25689818 PMC4537397

[B7] Brown S. A. McGue M. Maggs J. Schulenberg J. Hingson R. Swartzwelder S. (2008). A developmental perspective on alcohol and youths 16 to 20 years of age. Pediatrics 121, S290–S310. 10.1542/peds.2007-2243D 18381495 PMC2765460

[B8] Correa M. Salamone J. D. Segovia K. N. Pardo M. Longoni R. Spina L.. (2012). Piecing together the puzzle of acetaldehyde as a neuroactive agent. Neurosci. Bbiobehav. Rev. 36, 404–430. 10.1016/j.neubiorev.2011.07.009 21824493

[B9] Curran C. P. Marczinski C. A. (2017). Taurine, caffeine and energy drinks: reviewing the risks to the adolescent brain. Birth Defects Res. 109, 1640–1648. 10.1002/bdr2.1177 29251842 PMC5737830

[B10] Czachowski C. L. Samson H. H. (2002). Ethanol- and sucrose-reinforced appetitive and consummatory responding in HAD1, HAD2 and P rats. Alcohol. Clin. Exp. Res. 26, 1653–1661. 10.1111/J.1530-0277.2002.TB02467.X 12436053

[B11] Deng X. Deitrich R. A. (2008). Putative role of brain acetaldehyde in ethanol addiction. Curr. Drug Abuse Rev. 1, 3–8. 10.2174/1874473710801010003 19122804 PMC2613359

[B12] Dietze M. A. Kulkosky P. J. (1991). Effects of caffeine and bombesin on ethanol and food intake. Life Sci. 48, 1837–1844. 10.1016/0024-3205(91)90239-8 2041457

[B13] Doggett A. Qian W. Cole A. G. Leatherdale S. T. (2019). Youth consumption of alcohol mixed with energy drinks in Canada: assessing the role of energy drinks. Prev. Med. Rep. 14:100865. 10.1016/j.pmedr.2019.100865 31008029 PMC6458478

[B14] Dorofeikova M. V. Egorov A. Y. Filatova E. V. Orlov A. A. (2017). Sucrose-enriched diet during maturation increases ethanol preference in rats. Dokl. Biol. Sci. 475, 148–150. 10.1134/S0012496617040068 28861882

[B15] Ferré S. O’Brien M. C. (2011). Alcohol and caffeine: the perfect storm. J. Caffeine Res. 475, 153–162. 10.1089/jcr.2011.0017 24761263 PMC3621334

[B16] Garg A. Sharma A. Krishnamoorthy P. Garg J. Virmani D. Sharma T.. (2017). Role of niacin in current clinical practice: a systematic review. Am. J. Med. 130, 173–187. 10.1016/j.amjmed.2016.07.038 27793642

[B17] Gibson A. Woodside J. V. Young I. S. Sharpe P. C. Mercer C. Patterson C. C.. (2008). Alcohol increases homocysteine and reduces B vitamin concentration in healthy male volunteers–a randomized, crossover intervention study. QJM 101, 881–887. 10.1093/qjmed/hcn112 18790817 PMC2572692

[B18] Hansen A. W. Almeida F. B. Bandiera S. Pulcinelli R. R. Caletti G. Agnes G.. (2020). Correlations between subunits of GABA_A_ and NMDA receptors after chronic alcohol treatment or withdrawal and the effect of taurine in the hippocampus of rats. Alcohol 82, 63–70. 10.1016/j.alcohol.2019.08.005 31473305

[B19] Harada H. Kitazaki K. Tsujino T. Watari Y. Iwata S. Nonaka H.. (2000). Oral taurine supplementation prevents the development of ethanol-induced hypertension in rats. Hypertens. Res. 23, 277–284. 10.1291/hypres.23.277 10821139

[B20] Hipólito L. Sánchez M. J. Polache A. Granero L. (2007). Brain metabolism of ethanol and alcoholism: an update. Curr. Drug Metabol. 8, 716–727. 10.2174/138920007782109797 17979660

[B21] Iwata H. Matsuda T. Lee E. Yamagami S. Baba A. (1980). Effect of ethanol on taurine concentration in the brain. Experientia 36, 332–333. 10.1007/BF01952308 7189471

[B22] Jagim A. R. Harty P. S. Barakat A. R. Erickson J. L. Carvalho V. Khurelbaatar C.. (2022). Prevalence and amounts of common ingredients found in energy drinks and shots. Nutrients 14:314. 10.3390/nu14020314 35057494 PMC8780606

[B23] Julius U. (2015). Niacin as antidyslipidemic drug. Can. J. Physiol. Pharmacol. 93, 1043–1054. 10.1139/cjpp-2014-0478 26370906

[B24] Kunin D. Gaskin S. Rogan F. Smith B. R. Amit Z. (2000). Caffeine promotes ethanol drinking in rats. Alcohol 21, 271–277. 10.1016/s0741-8329(00)00101-4 11091031

[B25] Lees B. Kunin D. Gaskin S. Rogan F. Smith B. R. (2020). Association of prenatal alcohol exposure with psychological, behavioral and neurodevelopmental outcomes in children from the adolescent brain cognitive development study. Am. J. Psychiatry 177, 1060–1072. 10.1176/appi.ajp.2020.20010086 32972200 PMC7924902

[B26] Lemon C. H. Brasser S. M. Smith D. V. (2004). Alcohol activates a sucrose-responsive gustatory neural pathway. J. Neurophysiol. 92, 536–544. 10.1152/jn.00097.2004 14985409

[B27] Li Z. Zharikova A. Vaughan C. H. Bastian J. Zandy S. Esperon L.. (2010). Intermittent high-dose ethanol exposures increase motivation for operant ethanol self-administration: possible neurochemical mechanism. Brain Res. 1310, 142–153. 10.1016/j.brainres.2009.11.029 19944084 PMC2812672

[B28] López-Cruz L. Salamone J. D. Correa M. (2013). The impact of caffeine on the behavioral effects of ethanol related to abuse and addiction: a review of animal studies. J. Caffeine Res. 3, 9–21. 10.1089/jcr.2013.0003 24761272 PMC3643311

[B29] Marczinski C. A. Fillmore M. T. (2014). Energy drinks mixed with alcohol: what are the risks? Nutr. Rev. 72, 98–107. 10.1111/nure.12127 25293549 PMC4190582

[B500] May C. E. Haun H. L. Griffin W. C. III (2015). Sensitization and tolerance following repeated exposure to caffeine and alcohol in mice. Alcohol Clin. Exp. Res. 39, 1443–1452. 10.1111/acer.12794 26136115 PMC4515142

[B30] Messiha F. S. (1979). Taurine, analogues and ethanol elicited responses. Brain Res. Bull. 4, 603–607. 10.1016/0361-9230(79)90100-x 487215

[B31] Miyazaki A. Sano M. Fukuwatari T. Shibata K. (2012). Effects of ethanol consumption on the B-group vitamin contents of liver, blood and urine in rats. Br. J. Nutr. 108, 1034–1041. 10.1017/S0007114511006192 22172166

[B32] O’Brien M. C. Brien M. C. McCoy T. P. Rhodes S. D. Wagoner A. Wolfson M. (2008). Caffeinated cocktails: energy drink consumption, high-risk drinking and alcohol-related consequences among college students. Acad. Emerg. Med. 15, 453–460. 10.1111/j.1553-2712.2008.00085.x 18439201

[B33] Oh S. S. Ju Y. J. Park E. C. Jang S. I. (2019). Alcohol mixed with energy drinks (AmED) and negative alcohol-related consequences among South Korean college students. Int. J. Environ. Res. Public Health 16:1127. 10.3390/ijerph16071127 30934815 PMC6479579

[B34] Patrick M. E. Veliz P. Linden-Carmichael A. Terry-McElrath Y. M. (2018). Alcohol mixed with energy drink use during young adulthood. Addict. Behav. 84, 224–230. 10.1016/j.addbeh.2018.03.022 29734120 PMC5975232

[B35] Peacock A. Droste N. Pennay A. Miller P. Lubman D. I. Bruno R. (2015). Typology of alcohol mixed with energy drink consumers: motivations for use. Alcohol. Clin. Exp. Res. 39, 1083–1092. 10.1111/acer.12729 25988585

[B36] Potthoff A. D. Ellison G. Nelson L. (1983). Ethanol intake increases during continuous administration of amphetamine and nicotine, but not several other drugs. Pharmacol. Biochem. Behav. 18, 489–493. 10.1016/0091-3057(83)90269-1 6867054

[B501] Pulcinelli R. R. de Paula L. F. Nietiedt N. A. Bandiera S. Hansen A. W. Izolan L. R.. (2020). Taurine enhances voluntary alcohol intake and promotes anxiolytic-like behaviors in rats. Alcohol 88, 55–63. 10.1016/j.alcohol.2020.07.004 32698052

[B37] Quertemont E. De Neuville J. De Witte P. (1998). Changes in the amygdala amino acid microdialysate after conditioning with a cue associated with ethanol. Psychopharmacology (Berl) 139, 71–78. 10.1007/s002130050691 9768544

[B38] Quertemont E. Quertemont E. Dahchour A. Ward R. J. Witte P. (1999). Ethanol induces taurine release in the amygdala: an *in vivo* microdialysis study. Addict. Biol. 4, 47–54. 10.1080/13556219971830 20575769

[B39] Rada P. Avena N. M. Hoebel B. G. (2005). Daily bingeing on sugar repeatedly releases dopamine in the accumbens shell. Neuroscience 134, 737–744. 10.1016/j.neuroscience.2005.04.043 15987666

[B40] Rawat A. K. (1977). Effects of fructose and other substances on ethanol and acetaldehyde metabolism in man. Res. Commun. Chem. Pathol. Pharmacol. 16, 281–290. 847286

[B41] Rodríguez-Ortega E. Alcaraz-Iborra M. de la Fuente L. de Amo E. Cubero I. (2019). Environmental enrichment during adulthood reduces sucrose binge-like intake in a high drinking in the dark phenotype (HD) in C57BL/6J mice. Front. Behav. Neurosci. 13:27. 10.3389/fnbeh.2019.00027 30828291 PMC6384528

[B42] Roldán M. Echeverry-Alzate V. Bühler K.-M. Sánchez-Diez I. J. Calleja-Conde J. Olmos P.. (2018). Red Bull^®^ energy drink increases consumption of higher concentrations of alcohol. Addict. Biol. 23, 1094–1105. 10.1111/adb.12560 28940880

[B43] Samson H. Files F. Brice G. (1996). Patterns of ethanol consumption in a continuous access situation: the effect of adding a sweetener to the ethanol solution. Alcohol. Clin. Exp. Res. 20, 101–109. 10.1111/j.1530-0277.1996.tb01052.x 8651439

[B44] Scalese M. Denoth F. Siciliano V. Bastiani L. Cotichini R. Cutilli A.. (2017). Energy drink and alcohol mixed energy drink use among high school adolescents: association with risk taking behavior, social characteristics. Addict. Behav. 72, 93–99. 10.1016/j.addbeh.2017.03.016 28388494

[B45] Sharpe A. L. Samson H. H. (2003). Ethanol and sucrose self-administration components: effects of drinking history. Alcohol 29, 31–38. 10.1016/s0741-8329(02)00318-x 12657374

[B46] Simon M. Mosher J. (2007). Alcohol, energy drinks and youth: a dangerous mix. 1–18. Available online at: https://alcoholjustice.org/images/stories/EnergyDrinkReport.tif.

[B47] Squeglia L. M. Spadoni A. D. Infante M. A. Myers M. G. Tapert S. F. (2009). Initiating moderate to heavy alcohol use predicts changes in neuropsychological functioning for adolescent girls and boys. Psychol. Addict. Behav. 23, 715–722. 10.1037/a0016516 20025379 PMC2802333

[B48] Stratford T. R. Wirtshafter D. (2011). Opposite effects on the ingestion of ethanol and sucrose solutions after injections of muscimol into the nucleus accumbens shell. Behav. Brain Res. 216, 514–518. 10.1016/j.bbr.2010.08.032 20804790 PMC3245679

[B49] Suárez L. M. Solís J. M. (2006). Taurine potentiates presynaptic NMDA receptors in hippocampal Schaffer collateral axons. Eur. J. Neurosci. 24, 405–418. 10.1111/j.1460-9568.2006.04911.x 16836643

[B50] Takahashi T. T. Vendruscolo L. F. Takahashi R. N. (2015). Binge-like ingestion of a combination of an energy drink and alcohol leads to cognitive deficits and motivational changes. Pharmacol. Biochem. Behav. 136, 82–86. 10.1016/j.pbb.2015.07.007 26187003

[B51] Tarragon E. Calleja-Conde J. Giné E. Segovia-Rodríguez L. Durán-González P. Echeverry-Alzate V. (2021). Alcohol mixed with energy drinks: what about taurine?. Psychopharmacology (Berl) 238, 1–8. 10.1007/s00213-020-05705-7 33175215

[B52] Ulenius L. Adermark L. Söderpalm B. Ericson M. (2019). Energy drink constituents (caffeine and taurine) selectively potentiate ethanol-induced locomotion in mice. Pharmacol. Biochem. Behav. 187:172795. 10.1016/j.pbb.2019.172795 31669834

[B53] Verster J. C. Benson S. Johnson S. J. Alford C. Godefroy S. B. Scholey A. (2018). Alcohol mixed with energy drink (AMED): a critical review and meta-analysis. Hum. Psychopharmacol. 33:e2650. 10.1002/hup.2650 29417616 PMC5901036

[B54] Vieno A. Canale N. Potente R. Scalese M. Griffiths M. D. Molinaro S. (2018). The multiplicative effect of combining alcohol with energy drinks on adolescent gambling. Addict. Behav. 82, 7–13. 10.1016/j.addbeh.2018.01.034 29475135

[B55] Wang L. L. Yang A. K. He S. M. Liang J. Zhou Z. W. Li Y.. (2010). Identification of molecular targets associated with ethanol toxicity and implications in drug development. Curr. Pharm. Des. 16, 1313–1355. 10.2174/138161210791034030 20184550

[B56] Weldy D. L. (2010). Risks of alcoholic energy drinks for youth. J. Am. Board Fam. Med. 23, 555–558. 10.3122/jabfm.2010.04.090261 20616299

[B57] Wilson D. F. Matschinsky F. M. (2020). Ethanol metabolism: the good, the bad and the ugly. Med. Hypotheses 140:109638. 10.1016/j.mehy.2020.109638 32113062

[B58] Yildiz Z. D. Başaran-Küçükgergin C. Doǧru-Abbasoǧlu S. Uysal M. (2019). Protective effects of N-acetylcysteine and taurine on oxidative stress induced by chronic acetaldehyde administration in rat liver and brain tissues. Arch. Clin. Exp. Med. 4, 113–117. 10.25000/acem.579968

[B59] Zielińska A. Mazurek A. Siudem P. Kowalska V. Paradowska K. (2022). Qualitative and quantitative analysis of energy drinks using ^1^ H NMR and HPLC methods. J. Pharm. Biomed. Anal. 213:114682. 10.1016/j.jpba.2022.114682 35228055

